# Adipose-derived stromal cells improve functional recovery after spinal cord injury through TGF-β1/Smad3/PLOD2 pathway activation

**DOI:** 10.18632/aging.202399

**Published:** 2021-01-20

**Authors:** Fang Li, Hua Liu, Kun Zhang, Dong-Jie Xiao, Chang Wang, Yun-Shan Wang

**Affiliations:** 1 Cell Therapy Center, Jinan Central Hospital, Cheeloo College of Medicine, Shandong University, Jinan 250013, China; 2 Central Hospital Affiliated to Shandong First Medical University, Jinan 250013, China; 3 Shandong Research Center of Transplantation and Tissue, Jinan 250013, China; 4 Jinan Dien Forensic Judical Appraisal Institute, Jinan Central Hospital, Cheeloo College of Medicine, Shandong University, Jinan 250013, China

**Keywords:** adipose-derived stromal cells, spinal cord injury, cell transplantation, TGF-β1, PLOD2

## Abstract

Transplantation of mesenchymal stromal cells (MSCs) improves functional recovery in experimental models of spinal cord injury (SCI), but the mechanism is not fully understood. Activation of procollagen-lysine, 2-oxoglutarate 5-dioxygenase 2 (PLOD2), a collagen-modifying enzyme, reportedly follows MSC transplantation in an SCI animal model. We investigated the regulation of PLOD2 expression and its potential contribution to the neuroprotective effects of adipose-derived stromal cells (ADSCs) following mechanical injury to neurons *in vitro* and SCI *in vivo*. ADSCs enhanced wound healing *in vitro* and promoted functional recovery after their implantation near injury sites in a rat SCI model. These effects correlated with upregulation of PLOD2, MAP2, NSE and GAP43, and downregulation of GFAP, which is indicative of improved neuronal survival and axonal regeneration as well as reduced glial scar formation. The neurorestorative effect of ADSCs was weakened after inhibition of PLOD2 expression. ADSCs appeared to induce PLOD2 upregulation via TGF-β1 secretion, as ADSC-mediated PLOD2 expression, neuronal survival, and functional recovery after SCI were largely prevented by SB431542, a TGF-(1 receptor inhibitor. These findings indicate that ADSCs reduce lesion size and promote functional recovery after SCI mainly through activation of a TGF-β1/P-Samd3/PLOD2 pathway in spinal cord neurons.

## INTRODUCTION

Spinal cord injury (SCI) is a severe condition involving a variety of pathogenic factors that lead to structural and functional injury of the affected nerves, resulting in the loss of voluntary movements and sensation below the damaged plane [1]. Due to the limited regenerative capability of neuronal elements and its negative prognosis, developing an effective therapy for SCI remains an urgent clinical need.

Mesenchymal stromal cells (MSCs) are multipotent, tissue-specific stem cells that have garnered great interest for regenerative medicine [2]. It is generally accepted that the beneficial effects of MSCs are based on the secretion of a wide range of substances acting on host cells [3, 4]. Compared to bone marrow mesenchymal stromal cells (BMSCs), adipose-derived stromal cells (ADSCs) demonstrated a higher survival rate when transplanted into spinal cord injury sites in experimental SCI models *in vivo* [5]. Studies also reported that ADSCs secreted cytokines and growth factors which improved axonal regeneration and reduced cavity formation [6, 7]. However, despite the evidence supporting the use of ADSCs for SCI treatment, the specific mechanisms by which ADSCs promote spinal cord injury repair are not fully clear.

Torres-Espín et al. reported that MSC transplantation after SCI enhanced the expression of procollagen-lysine, 2-oxoglutarate 5-dioxygenase 2 (PLOD2), an enzyme that contributes to extracellular matrix (ECM) organization by catalyzing the formation of collagen crosslinks [8]. PLOD2 is mainly located in the rough endoplasmic reticulum, shows wide tissue distribution, and has been demonstrated to enhance cancer cell motility and metastasis via the PI3K-AKT or TGF-β/Smad2 signaling pathways [9–12]. However, the regulation and role of PLOD2 on SCI remain uncertain. To address this issue, we established a cell-based mechanical injury model and a rat SCI model to explore and characterize the mechanism by which ADSCs induce neuronal PLOD2 expression to mediate neuroprotection.

## RESULTS

### Characterization of ADSCs

**Figure 1:**
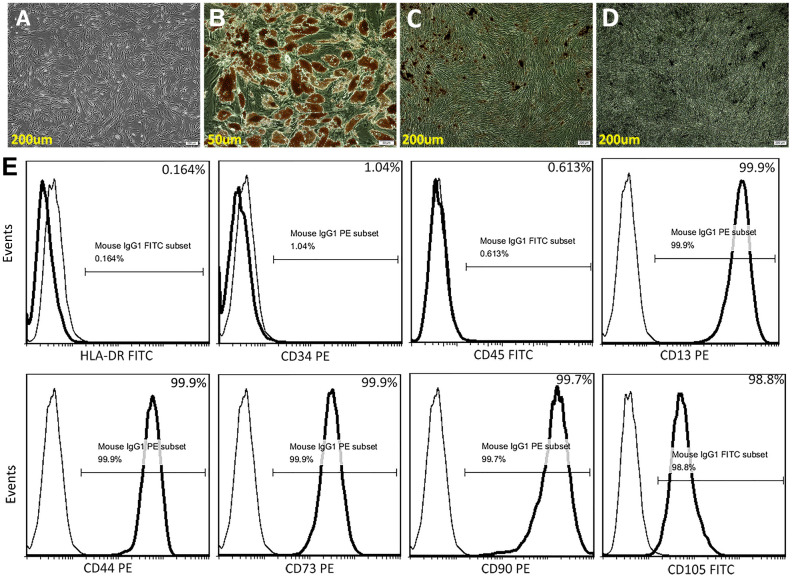
**Characterization of ADSCs.** (**A**) Microscopic image showing spindle-shaped, third-passage ADSCs. (**B**) Oil red O staining for adipocytes. (**C**) Alizarin Red S staining for osteocytes. (**D**) Von Kossa staining for osteocytes. (**E**) Human ADSC characterization using flow cytometry. Isolated ADSCs showed high expression of CD13, CD44, CD73, CD90, and CD105 (positive) and very low expression of HLA-DR, CD34, and CD45 (negative).

ADSCs *in vitro* exhibited a fibroblast-like morphology (Figure 1A), and differentiated into adipocytes and osteocytes, respectively, after 3 weeks of adipogenic and osteogenic induction (Figure 1B–1D). Flow cytometry showed that undifferentiated ADSCs expressed high levels of CD13, CD44, CD73, CD90 and CD105, and were negative for the hematopoietic stem cell markers HLA-DR, CD34, and CD45 (Figure 1E).

### ADSCs promote neuronal recovery *in vitro*

**Figure 2:**
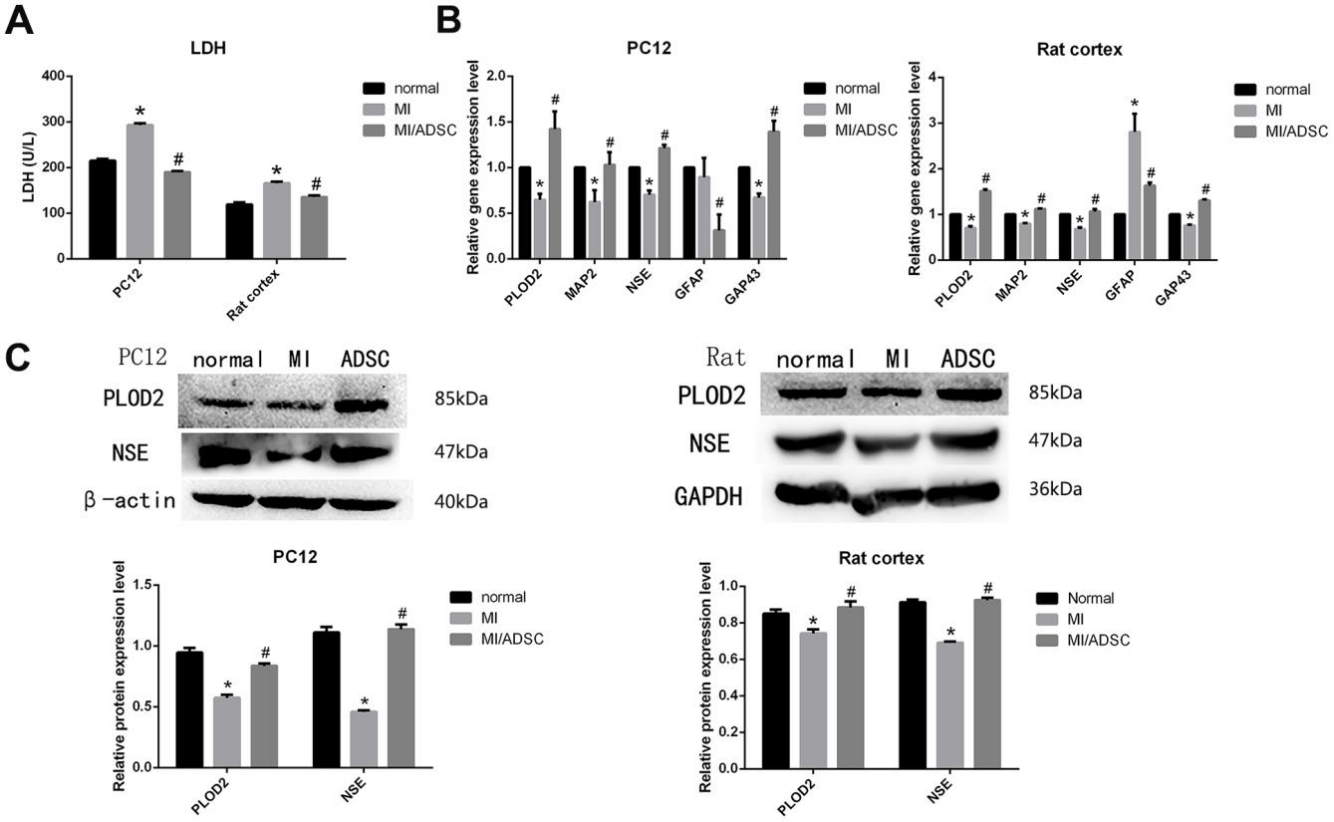
**ADSC co-culture promotes survival of mechanically injured neurons.** (**A**) LDH release assay results from PC12 and primary rat neurons exposed to MI. (**B**) Real-time PCR analysis of PLOD2, MAP2, NSE, GAP43, and GFAP in MI-treated cells. (**C**) Western-blot analysis showing increased expression of PLOD2 and NSE in MI-treated cells co-cultured with ADSCs. Results presented as mean ± SD and evaluated with one-way ANOVA. *P<0.05, compared to control (no MI); #*P*<0.05, compared to MI. MI: mechanical injury.

To investigate the influence of ADSCs on neuronal recovery from injury, a neuron- ADSC co-culture system was first established *in vitro*. A wound-healing assay involving mechanical injury (MI) was then performed on both differentiated PC12 cells and rat cortical neurons. Results showed that wound closure rates were significantly higher in cells co-cultured with ADSCs (plated on Transwell inserts) for 72 h (Supplementary Figure 1A). Moreover, EdU assays showed that co-cultured ADSCs enhanced the proliferation of rat cortical neurons (Supplementary Figure 1B). Accordingly, lactate dehydrogenase (LDH) release assay results indicated that neuronal damage induced by MI was reversed by ADSC co-culturing (Figure 2A).

We next conducted qRT-PCR and western blot assays, which showed that the downregulation of the neuronal markers MAP2 and NSE induced by MI was prevented upon ADSC co-culture (Figure 2B, 2C). Moreover, ADSCs also promoted axonal regeneration, denoted by increased expression of GAP43 in co-cultured neurons (Figure 2B) [13]. Interestingly, decreased expression of the glial cell marker GFAP was also observed in neurons co-cultured with ADSCs (Figure 2B). These results suggest that ADSCs improve neuronal survival, promote axonal regeneration, and reduce glial scar formation after MI.

### PLOD2 inhibition impairs ADSCs’ neuroprotective effects

A previous study determined that PLOD2 expression was increased in the injured spinal cord after treatment with MSCs or olfactory ensheathing cells [8]. In line with these data, our results indicated that co-culture with ADSCs reversed the decrease in neuronal PLOD2 expression caused by MI (Figure 2B, 2C). To investigate whether ADSC-mediated neuronal recovery from MI is mediated by PLOD2 upregulation, a PLOD2 inhibitor, minoxidil, was added into the co-culture system. Based on qRT-PCR and western blot analyses, we selected an experimental concentration of 0.5 mM and 48-h exposure for minoxidil treatment (Supplementary Figure 2).

**Figure 3:**
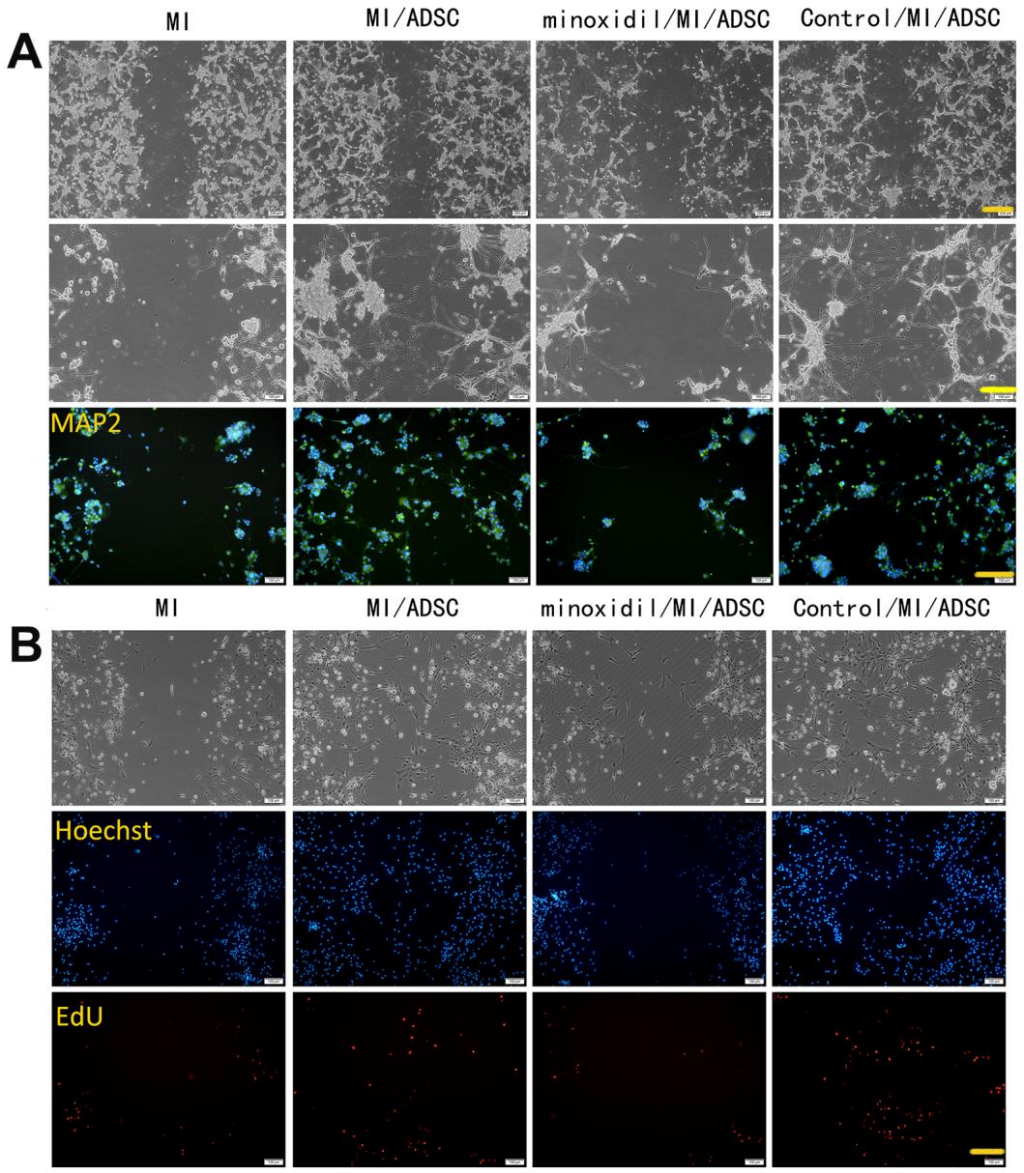
**PLOD2 inhibition attenuates ADSC-mediated neuronal protection following mechanical injury *in vitro*.** (**A**) Light microscopy and MAP2 immunofluorescence imaging of PC12 cells 3 days after ADSC co-culture. Scale bars: 200 μm (upper row), 100 μm (middle and lower rows). (**B**) EdU assay results showing enhanced proliferation of rat cortical neurons after ADSC co-culture and reduced proliferation in the presence of the PLOD2 inhibitor minoxidil. Scale bar: 100 μm.

**Figure 4:**
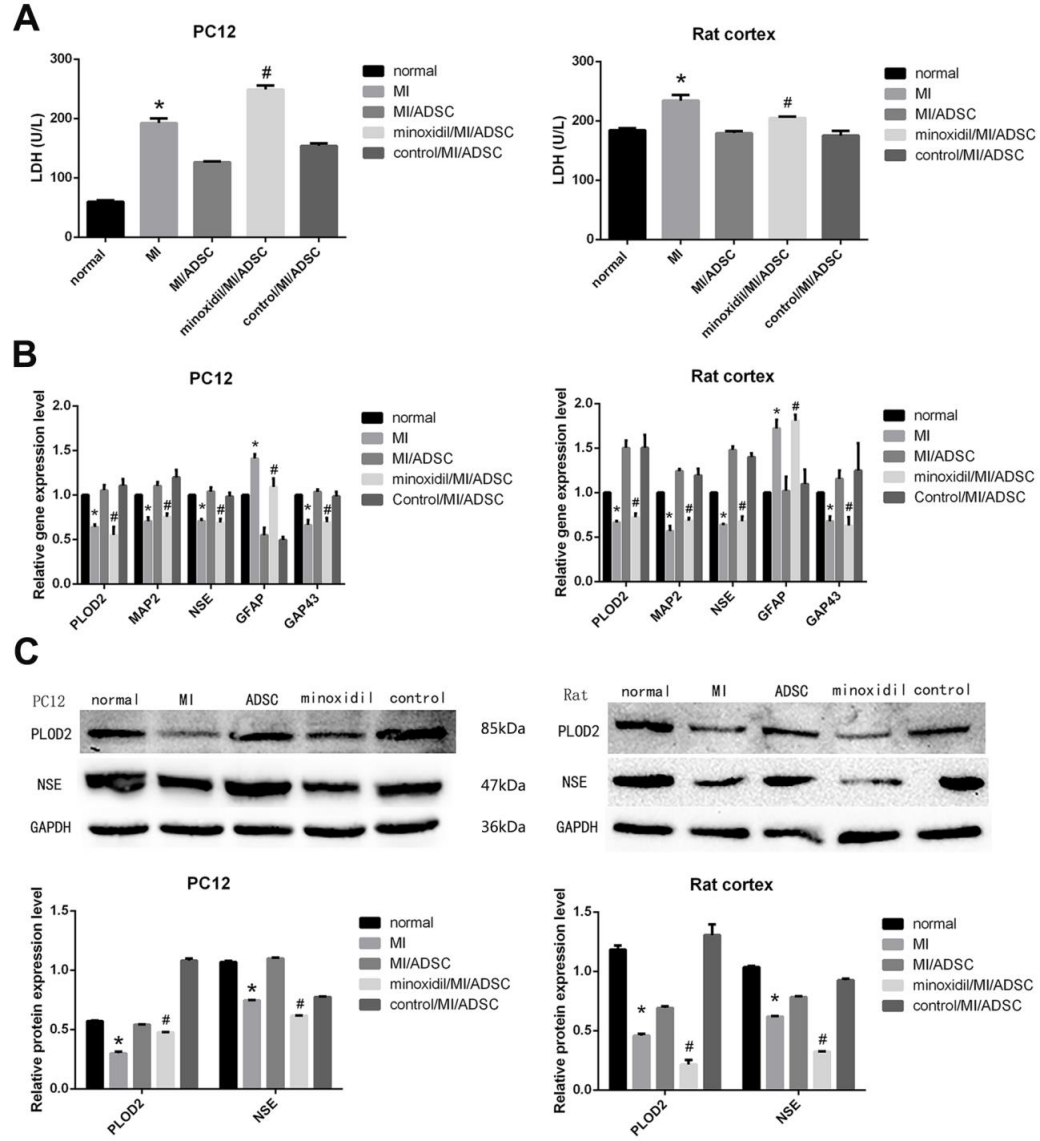
**Inhibited the expression of PLOD2 by minoxidil, the neurorestorative effect of ADSCs was weakened.** (**A**) LDH release assay result from MI-exposed cells co-cultured with ADSCs in the presence or absence of minoxidil. (**B**) Analysis of PLOD2, MAP2, NSE, GAP43, and GFAP transcript expression in MI-treated neurons co-cultured with ADSCs in the presence or absence of minoxidil. (**C**) Western blot analysis of PLOD2 and neuronal markers. Results presented as mean ± SD and evaluated with one-way ANOVA. *P<0.05, compared to control (no MI) and MI/ADSC groups; #*P*<0.05, compared to MI/ADSC and control (DMSO/MI/ADSC) groups.

Both microscopic observations and MAP2 immunofluorescence showed that the neurorestorative effect of ADSCs was weakened upon inhibition of PLOD2 expression (Figure 3A). In turn, EdU assay results showed that minoxidil reduced the stimulatory effect of ADSCs on the proliferation of rat cortical neurons (Figure 3B), while LDH release assays indicated that ADSC-mediated neuronal protection following MI was attenuated by minoxidil application (Figure 4A). Moreover, both qRT-PCR and western blotting confirmed that minoxidil exposure inhibited PLOD2 expression in both PC12 cells and rat cortical neurons, and attenuated or prevented the changes in MAP2, NSE, and GAP43 expression observed upon ADSC co-culture (Figure 4B, 4C).

### ADSCs exert neuronal repair functions mainly through the TGF-β1/Smad3 signaling pathway

**Figure 5:**
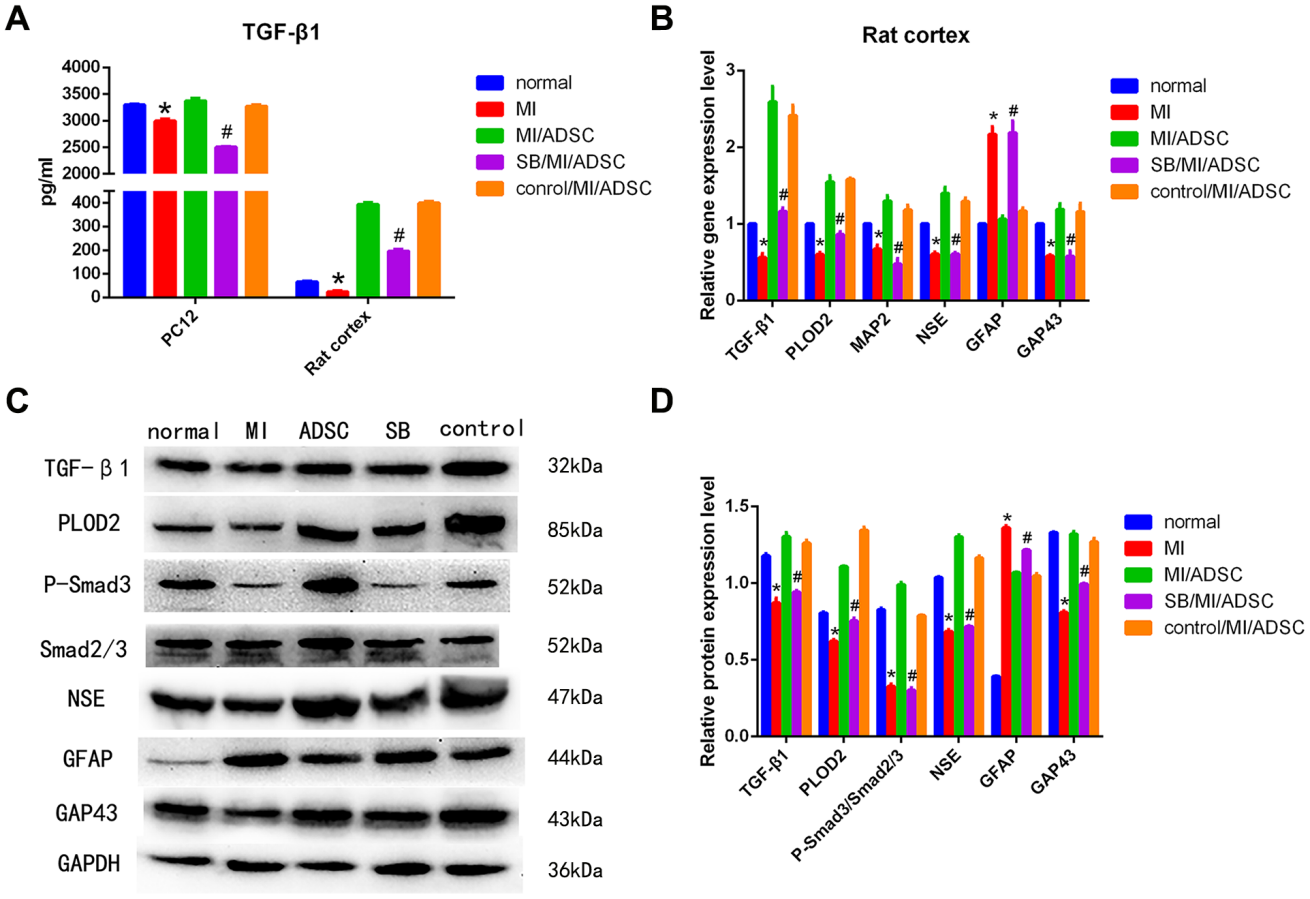
**ADSCs protect neurons from MI by activating the TGF-β1/Smad3 signaling pathway.** (**A**) Analysis of TGF-β1 secretion in culture supernatants from cells subjected to MI and treated with SB431542 (SB). (**B**) Detection of TGF-β1, PLOD2, MAP2, NSE, GFAP, and GAP43 mRNA levels by qRT-PCR. (**C**, **D**) Western blot analysis of TGF-β1, PLOD2, P-Smad3, Smad2/3, and neuronal/glial markers in rat cortical neurons. Results presented as mean ± SD and evaluated with one-way ANOVA. *P<0.05, compared to normal and MI/ADSC groups; #*P*<0.05, compared to MI/ADSC and control groups.

To elucidate the molecular mechanism(s) that mediate ADSC-induced PLOD2 upregulation, we explored potential changes in the expression of TGF-β1, an important MSC-secreted cytokine that was shown to alleviate SCI [14]. ELISA results showed that TGF-β1 levels were significantly increased in cell culture supernatants from both PC12 cells and rat cortical neurons upon ADSC co-culture (Figure 5A).

Then, we assessed whether ADSC-mediated PLOD2 upregulation in neurons subjected to MI is affected by TGF-β1 inhibition with SB431542 (SB). As expected, ELISA results showed that the expression of TGF-β1 was significantly decreased in the SB/MI/ADSC group, compared with the MI/ADSC and the dimethyl sulfoxide (DMSO) control groups (Figure 5A). In turn, both qRT-PCR and western blot analyses showed that the expression of TGF-β1, P-Smad3, and PLOD2 was significantly decreased in the SB/MI/ADSC group, compared to the MI/ADSC and the DMSO control groups (Figure 5B–5D). These analyses also showed that the expression of MAP2, NSE, and GAP43 was significantly decreased, while GFAP levels were significantly increased, upon SB/MI/ADSC treatment (Figure 5B–5D). We thus concluded that the neurorestorative effect of ADSCs is due to an increase in PLOD2 expression induced by TGF-(1 stimulation and Smad3 activation.

**Figure 6:**
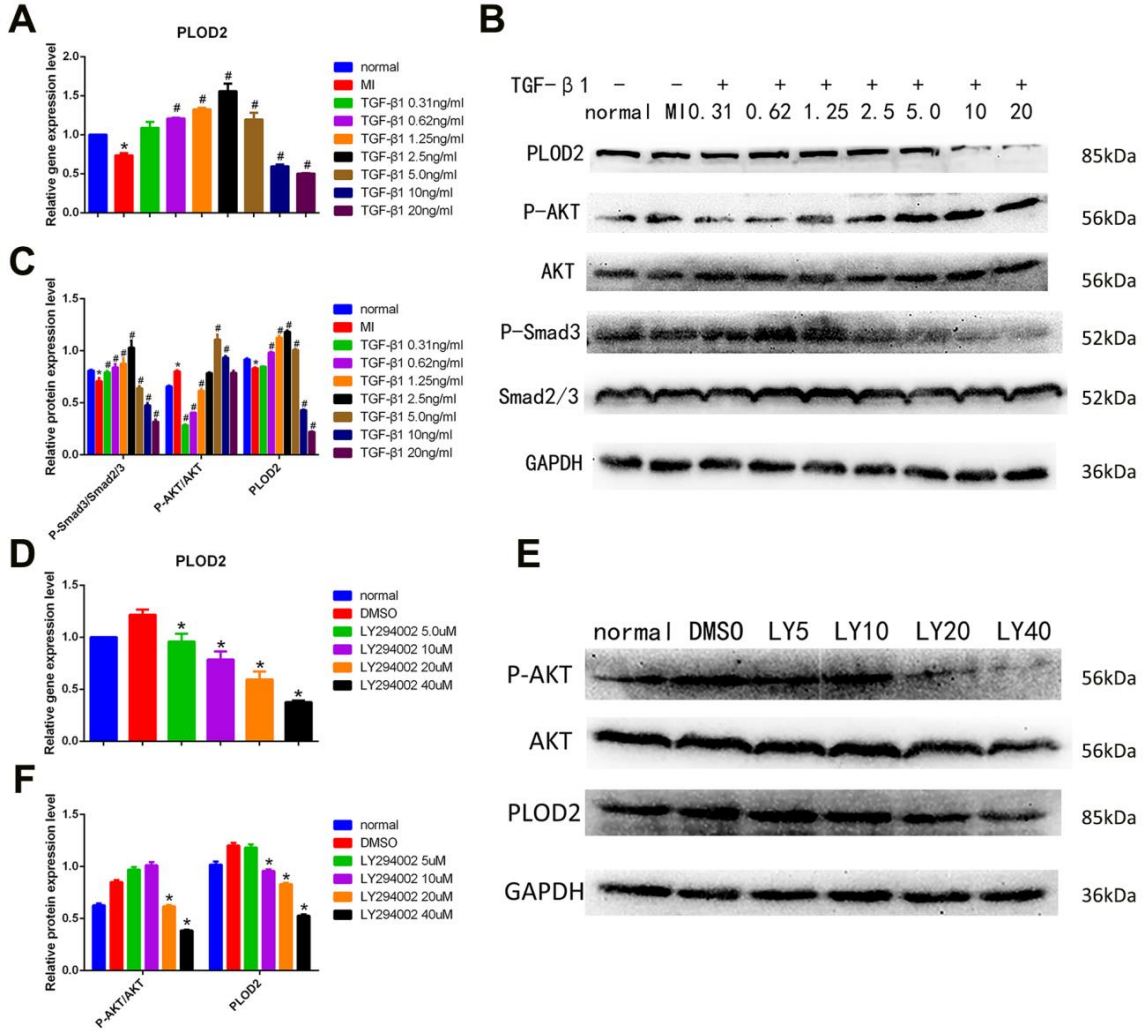
**TGF-β1/Smad3 pathway activation mediates ADSC-induced PLOD2 expression in neurons *in vitro*.** (**A**) Analysis of PLOD2 expression via qRT-PCR in PC12 cells treated with different concentrations of TGF-β1. (**B**, **C**) Western blot analysis of PLOD2, P-AKT, AKT, P-Smad3, and Smad2/3 expression in PC12 cells treated with different concentrations of TGF-β1. (**D**) Analysis of PLOD2 expression via qRT-PCR in PC12 cells treated with LY294002. (**E**, **F**) Western blot analysis of P-AKT, AKT, and PLOD2 expression in LY294002-treated PC12 cells. Results presented as mean ± SD and evaluated with one-way ANOVA. *P<0.05, compared to normal and DMSO groups; #*P*<0.05, compared to MI. LY5: LY294002 (5μM).

Next, we analyzed the effect of exogenous TGF-β1 on gene expression in PC12 cells subjected to MI. Results from qRT-PCR assays showed that low TGF-β1 concentrations (0.31-2.5 ng/ml) promoted the expression of PLOD2; this effect was weakened at 5.0 ng/ml, and inhibited instead at TGF-β1 concentrations of 10 and 20 ng/ml (Figure 6A). Since PLOD2 has been reported to be positively regulated by PI3K-AKT signaling pathway activation in cancer cells [10], we used western blotting to evaluate P-AKT and P-Smad3 expression in PC12 cells. We found that low concentrations of TGF-β1 inhibited the expression of P-AKT and promoted the expression of P-Smad3, while high TGF-β1 concentrations had the opposite effect (Figure 6B–6C). These observations suggest that ADSCs mediate neuroprotective effects by releasing TGF-β1, which promotes neuronal PLOD2 expression through activation of TGF-β1/Smad3 signaling.

Next, we used the PI3K/AKT inhibitor LY294002 to further assess whether PLOD2 expression is influenced by PI3K/AKT signaling in PC12 cells. qRT-PCR and western blotting results showed that at LY294002 concentrations ≥20 μM the expression of P-AKT was significantly inhibited, while the expression of PLOD2 decreased at the same time (Figure 6D–6F). These results confirmed that although P-AKT positively regulates PLOD2 expression in PC12 cells, in the presence of ADSCs PLOD2 upregulation is mainly elicited by activation of the TGF-β1/Smad3 signaling pathway.

### TGF-β1 mediates the therapeutic effects of ADSCs in a rat model of SCI

**Figure 7:**
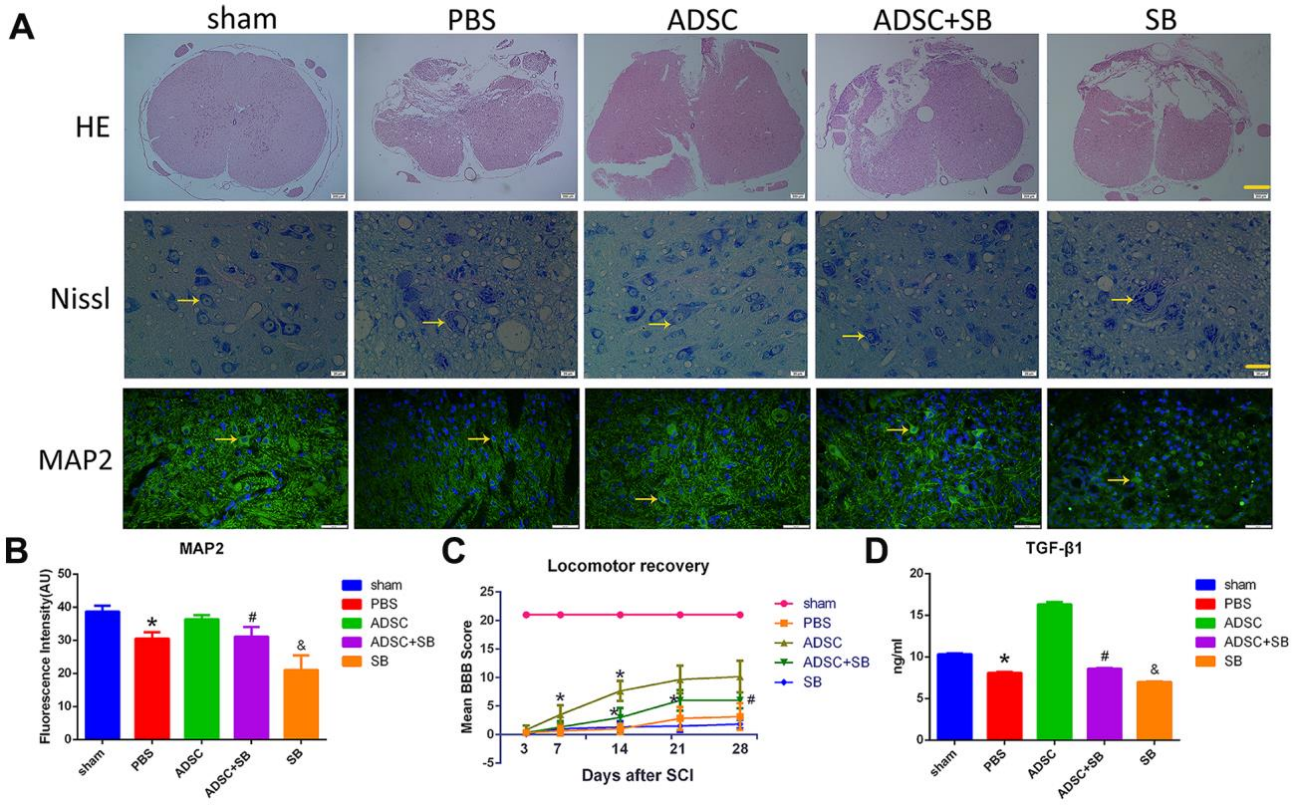
**Inhibition of TGF-β1/Smad3 signaling reverses the therapeutic effect of ADSCs on SCI.** (**A**) H&E staining images depicting pathological changes in the rat spinal cord following experimental SCI. Scale bar: 200 μm. Nissl staining analysis of residual neurons in transverse spinal cord sections 3-5 mm away from the injury site. Scale bar: 20 μm. Immunofluorescence analysis of MAP2 expression in injured spinal cord tissue showing reduced neuronal injury after ADSC transplantation. Scale bar: 50 μm. All the samples were obtained 3 days after ADSC transplantation. (**B**) MAP2 immunofluorescence in injured spinal cord tissue. The increase in MAP2 expression induced by ADSCs transplantation was attenuated by SB431542 treatment. (**C**) Assessment of locomotor behavior (BBB scale) after spinal cord injury (n=6/group). Results are mean ± SD; differences were evaluated with one-way ANOVA and t-test. *P<0.05 vs 3 days, #*P*<0.05, ADSC vs ADSC+SB. (**D**) ELISA analysis of rat serum TGF-β1 levels 3 days after ADSC transplantation/SB administration. *P<0.05, compared to sham and ADSC groups; #*P*<0.05, compared to ADSC and SB groups; &*P*<0.05, compared to PBS and ADSC+SB groups.

Our *in vitro* research showed that ADSCs activated the expression of PLOD2 mainly through the TGF-β1/Smad3 signaling pathway to exert neuronal repair functions. To determine whether a similar mechanism operates *in vivo*, a rat SCI model was established. Hematoxylin and eosin (H&E) and Nissl staining showed that SB431542 administration reversed the reduction in lesion volume induced by ADSC transplantation and decreased the formation of Nissl bodies at injured sites (Figure 7A). Furthermore, immunofluorescence analysis of MAP2 expression in spinal cord tissue showed that ADSC transplantation mitigated neuronal injury, and this effect was reduced by SB431542 (Figure 7A, 7B).

In turn, assessment of post-injury motor behavior using the Basso, Beattie, and Bresnahan (BBB) scale showed accelerated locomotor recovery in ADSC-treated rats, while marked attenuation of this effect was evident on days 7 and 14 post-SCI in rats treated with SB431542 (Figure 7C). Of note, no significant differences in locomotor function were detected after 21 days ADSCs treatment following SCI. This indicates that under our experimental settings, the beneficial effects of ADSCs on SCI are relatively short-lasting.

**Figure 8:**
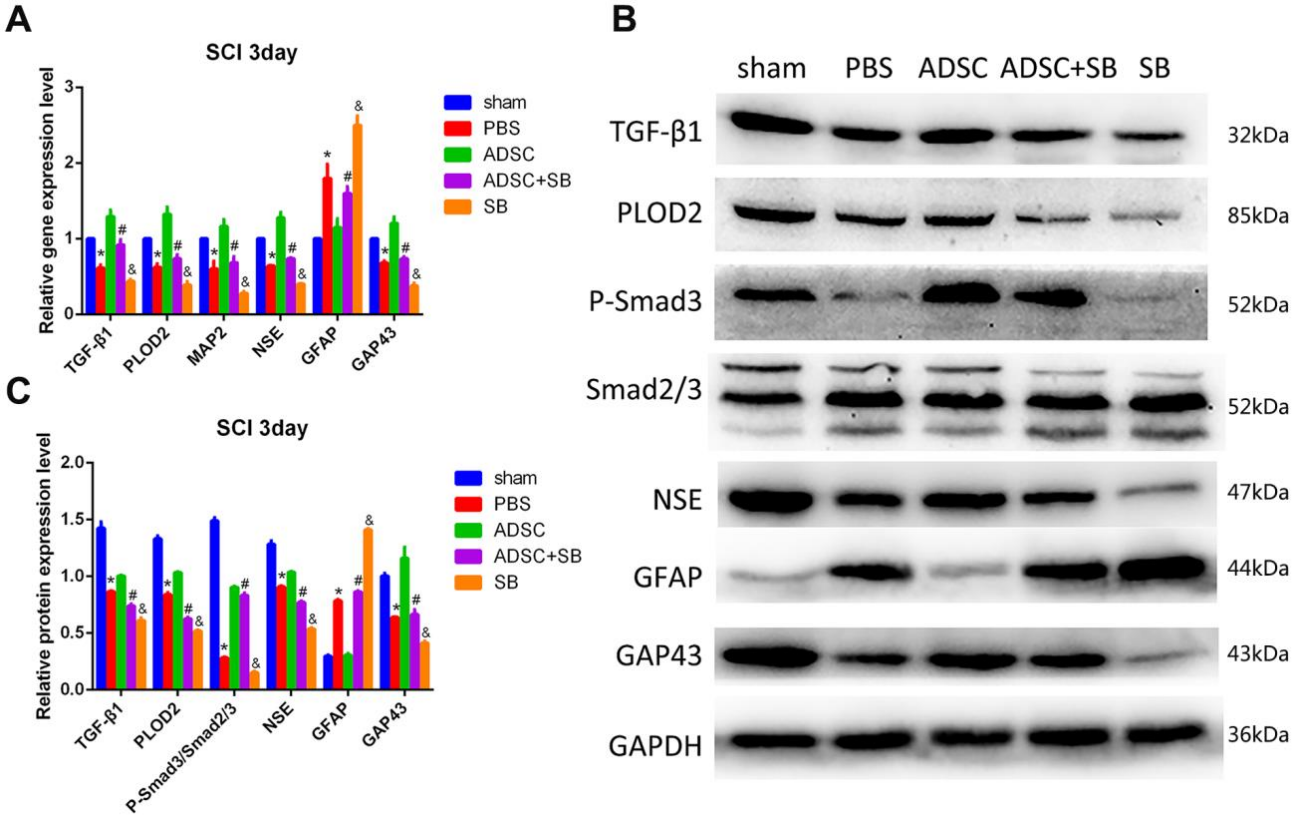
**Molecular changes associated with ADSC transplantation in the rat SCI model.** (**A**) Analysis of TGF-β1, PLOD2, MAP2, NSE, GAP43, and GFAP via qRT-PCR in spinal cord samples. (**B**, **C**) Western blot analysis of spinal cord samples. Results presented as mean ± SD and evaluated with one-way ANOVA. *P<0.05, compared to sham and ADSC groups; #*P*<0.05, compared to ADSC and SB groups; &*P*<0.05, compared to PBS and ADSC+SB groups.

**Figure 9:**
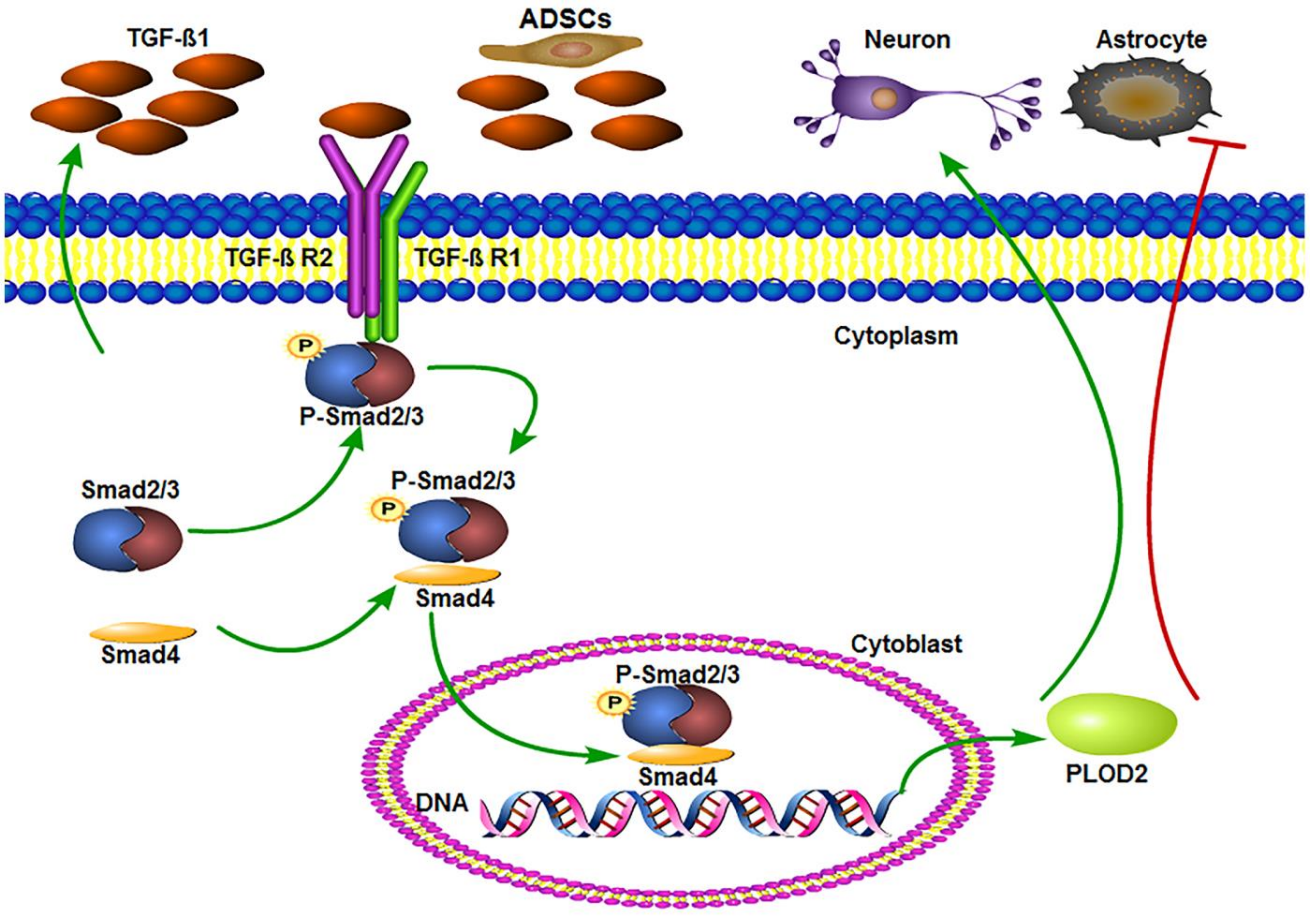
**ADSCs promote functional recovery after spinal cord injury by activating the TGF-β1/Smad3/PLOD2 signaling pathway.** The TGF-β1 complex phosphorylates Smad2/3, which bind to Smad4 and translocate to the nucleus to promote the expression of PLOD2. PLOD2 upregulation creates a favorable environment for neuronal survival and axonal regeneration, limiting also glial scar formation.

Meanwhile, ELISA results showed increased serum levels of TGF-β1 in ADSC-implanted rats, an effect that was significantly reversed by co-administration of SB431542 (Figure 7D). In turn, qRT-PCR and western blot assays showed upregulation of TGF-β1, P-Smad3, PLOD2, MAP2, NSE, and GAP43, and downregulation of GFAP expression in SCI samples 3 days after ADSC transplantation. These changes were reversed, however, following SB431542 treatment (Figure 8A–8C). Consistent with our *in vitro* results, these data demonstrate that ADSCs promote functional recovery in SCI rats through the release of TGF-β1 and stimulation of PLOD2 expression by spinal cord cells. This creates a favorable environment for the regeneration of neurons and axons, while reducing glial scar development (Figure 9).

## DISCUSSION

Research on SCI treatments typically encompasses two main aspects, neuroprotection and regeneration. Because of the presence of the blood-spinal cord barrier, the intrinsically limited regenerative capacity of mature neurons, and the accumulation of several factors that inhibit nerve regeneration, the treatment of SCI continues to represent a difficult medical challenge [15]. Studies have demonstrated that transplantation of MSCs improves functional recovery in experimental models of SCI [16, 17]. Likewise, self-derived ADSCs were shown to secrete growth factors promoting functional recovery, evidenced by enhanced axonal regeneration and tissue vascularization, and decreased inflammatory cell infiltration into the lesion site, in rat models of SCI [5, 18, 19]. Our study demonstrated that ADSCs promote the recovery of injured neurons in a co-culture system and reduce neuronal release of LDH. Meanwhile, *in vivo* transplantation of ADSCs into lesion sites following experimental SCI in rats led to reduced lesion volume, upregulation of neuronal/axonal markers, decreased glial reactivity, and transient but significant improvement in locomotor behavior.

Our data suggested that upregulation of neuronal PLOD2 expression following MI *in vitro* and SCI *in vivo* is a key pro-survival signal triggered by the proximity of ADSCs. As an important modulator of fibrotic collagen, PLOD2 has been shown to promote tumor metastasis directly, by enhancing tumor cell migration, and indirectly, by inducing collagen reorganization [20–23]. PLOD2 is regulated by HIF-1α, TGF-β1, and microRNA-26a/b through the PI3K-AKT or the TGF-β/Smad signaling pathways in tumor cells [24–27]. Our results demonstrated that co-culture with ADSCs increases the expression of PLOD2 in injured neurons. When PLOD2 expression was inhibited, the expression of LDH was increased and the expression of MAP2, NSE, and GAP43 was significantly decreased. These results indicated that ADSCs mediate PLOD2 upregulation to promote spinal neuron regeneration following SCI.

Our experiments showed that the neuronal protection conferred by ADSCs via PLOD2 upregulation is mediated by secretion of TGF-β1, a multifunctional cytokine with anti-inflammatory, reparative, and neuroprotective functions [28, 29]. TGF-β1 seems to play a complex role in tissue repair after SCI. Some studies suggested that TGF-β1/P-Smad2 pathway activation promotes neurite outgrowth and functional recovery after SCI [14], while others indicated that TGF-β suppression alleviates SCI by reducing oxidative stress and inflammation [30]. Our results showed that TGF-β1 expression was decreased in MI and SCI, and enhanced, albeit modestly, both *in vivo* and *in vitro* in the presence of ADSCs. Accordingly, we found that low concentrations of TGF-β1 inhibited the expression of P-AKT and promoted instead the expression of both P-Smad3 and PLOD2 in cultured neurons, while high concentrations of TGF-β1 had the opposite effect. Although these findings may help explain the controversy surrounding the effects of TGF-β on SCI, more research is needed to elucidate whether dose-dependent mechanisms impact TGF-β-mediated activation of alternative signaling pathways, particularly those involving AKT.

The neuroregenerative effect of TGF-β1/Smad3 pathway activation on SCI was confirmed through experiments that showed that SB431542, and ALK5/TGF-β type I receptor inhibitor, prevented the expression of P-Smad3 both *in vitro* and in our rat SCI model *in vivo*. Consequently, neuronal recovery from MI was inhibited after SB431542 treatment, while locomotor activity was impaired in ADSC-transplanted animals that received the inhibitor. Importantly, the expression of PLOD2 was also inhibited by SB431542 both *in vitro* and *in vivo*. We can thus conclude that the neurorestorative effect of ADSCs is mediated by TGF-(1 secretion, leading to activation of TGF-(1/Smad3 signaling and PLOD2 upregulation in neurons.

Although we did not directly address the role of PLOD2 in neuronal survival and axonal regeneration following MI or SCI, available evidence suggest that modification and reorganization of collagen fibers may be involved in these phenomena [21]. Collagen is crucial for cell-cell and cell-extracellular matrix (ECM) signaling and adhesion, i.e. events that support ADSC viability and affect the stability and availability of growth factors involved in neuronal survival, differentiation, and axonal outgrowth [31]. Recently, Belal Neyazi et al. reported for the first time the expression of PLOD2 in brain arteriovenous malformations (bAVM) and suggested a potential role for PLOD2 in bAVM pathophysiology [32]. Therefore, in future studies we will seek to determine whether PLOD2 can modulate ECM signaling to promote neurite outgrowth and functional recovery after SCI.

## MATERIALS AND METHODS

### Culture and identification of ADSCs

Approval for human tissue sample collection was obtained from the Ethics Committee of Jinan Central Hospital. Informed consent was obtained from all subjects prior to the study. ADSCs were isolated from 50-mL volumes of human adipose tissue obtained by abdominal liposuction surgery. Tissues were washed three times with phosphate-buffered saline (PBS) to remove red blood cells. After measuring the lipid fraction volume, an equal amount of collagenase I (0.2% w/v) solution was added for 30 min to digest the tissue at 37° C in a shaker set at 200 rpm. The resulting cell suspension was filtered using a 70 μm cell strainer to eliminate the undigested fragments and cultured in Alpha Minimum Essential Medium (α-MEM, Gibco, Grand Island, NY, USA) at 37° C and 5% CO2 [33, 34]. ADSCs were identified according to standard criteria [35] and used on passages 3-5 for experiments.

Differentiation of ADSCs into osteogenic and adipogenic lineages was carried out using a differentiation medium kit (Cyagen, Guangzhou, China). After 3 weeks, Alizarin Red S, Von Kossa and Oil red O staining was used to identify osteoblasts and adipocytes.

Characterization experiments were conducted by incubating ADSCs with monoclonal PE-conjugated antibodies against CD13, CD34, CD44, CD73, and CD90 or with FITC-conjugated antibodies against HLA-DR, CD45, and CD105 (BD Pharmingen, San Diego, CA, USA) for 20 min at room temperature. Cell fluorescence was evaluated on a FACSCalibur flow cytometer (Becton Dickson, Franklin Lakes, NJ, USA) and data was analyzed using FlowJo software.

### Cell culture

PC12 cells were purchased from the Stem Cell Bank of the Chinese Academy of Sciences and maintained in F12K Medium supplemented with 10% horse serum, 8% fetal bovine serum (FBS), 100 U/ml of penicillin, and 100 U/ml of streptomycin (Gibco, Waltham, MA, USA) in a humidified incubator containing 5% CO2 at 37° C. Differentiation was induced by incubation in medium containing 150 ng/ml NGF for 6 days before subsequent experiments. Primary neurons were extracted from the cortex of newborn Wistar rats and cultured in Neurobasal medium with 1% B27. After 6 days, neurons were used for downstream experiments.

### Mechanical injury assay and cell co-culturing

A mechanical injury (MI) assay was used to evaluate wound healing *in vitro*. Briefly, PC12 cells and rat cortical neurons were independently seeded on 6-well plates. Upon confluence, the cultures were scratched at the midline with a 10-μl pipette tip, rinsed with PBS, and replenished with regular media. ADSCs were inoculated onto transwells (3×10^5^ cells per well) and co-cultured with injured neurons for 3 days. In separate experiments, following MI PC12 cells were treated for 3 days with 0.31, 0.62, 1.25, 2.5, 5.0, 10, or 20 ng/ml TGF-β1 (PeproTech, Rocky Hill, NJ, USA).

### Immunofluorescence

Cells cultured on 24-well plates and frozen sections of spinal cord tissue were fixed with 4% paraformaldehyde for 20 min. The samples were then permeabilized using 0.5% Triton X-100 for 20 min and blocked with sheep serum blocking solution for 30 min at room temperature. Primary antibodies against MAP2 (1:100, Abcam, Cambridge, UK) were added at 4° C overnight, followed by incubation with Alexa Fluor 488-conjugated goat anti-rabbit secondary antibody for 1 h at room temperature. DAPI was used to counterstain cell nuclei [28]. Fluorescence images were captured under a fluorescence microscope (Olympus BX-53, Tokyo, Japan), and Image J software was used to perform measurements.

### Cell proliferation assay

Following MI, cell proliferation was measured using an EdU staining kit (RIBOBIO, Guangzhou, China). Fluorescence microscopy was used for data quantification.

### LDH release assay

Cell injury was confirmed by measuring the amount of LDH released [36]. Following treatments, cell supernatants were collected and centrifuged at 12,000 rpm for 5 min. An LDH assay kit (Nanjing Jiancheng Bioengineering Institute, Nanjing, China) was used as per the vendor’s specifications, with absorbance measured at 450 nm.

### TGF-β1 measurement

Cell culture supernatants and rat blood sera were collected three days after ADSC treatment, stored at - 80° C, and tested within one month. TGF-β1 levels were measured in triplicate using a Rat TGF-β1 ELISA Kit (Elabscience, Wuhan, China) following manufacturer’s instructions.

### Quantitative real-time PCR

Total RNA was isolated from cells and tissues using TRIzol reagent, after which mRNA (1 μg) was reverse transcribed to cDNA using PrimeScript RT Master Mix (Takara Bio, Tokyo, Japan). Transcript expression was assessed by quantitative PCR using an Applied Biosystem 7500 Real-Time PCR System (Thermo Fisher Scientific, USA). Target (rat TGF-β1, PLOD2, MAP2, NSE, GFAP, and GAP43) cDNA amplification was measured using SYBR Premix Ex Taq II (Takara Bio, Japan). Fold-change expression for each target mRNA was calculated with the CT (2^-ΔΔCT^) method, using β-actin levels for normalization. PCR assays were performed at least 3 times. Primer sequences are listed in Supplementary Table 1.

### Western blotting

Whole-cell lysates from 0.5-cm sections of spinal cord tissue containing the injury epicenter, cortical neurons, and PC12 cells were prepared in radioimmunoprecipitation assay (RIPA) lysis buffer. Protein samples (50 μg) were separated by 10% SDS-PAGE, transferred to PVDF membranes, and incubated with primary antibodies against TGF-β1 (1:500, Abcam, UK), PLOD2 (1:500, Proteintech, Chicago, IL, USA), P-Smad3 (1:300, Cell Signaling Technology, Boston, USA), Smad2/3 (1:1,000, Cell Signaling Technology, USA), P-AKT (1:5,000, Abcam, UK), AKT (1:10,000, Abcam, UK), NSE (1:2,000, Proteintech), GFAP (1:500, Abcam, UK), GAP43 (1:1,000, Abcam, UK) and GAPDH (1:2,500, Proteintech, USA) at 4° C overnight. The next day, the membranes were incubated with corresponding secondary antibodies (1:10,000 dilution) at room temperature for 1 h [37]. Immunoreactive bands were visualized by enhanced chemiluminescence (ECL) and band density quantified using Image J software.

### SCI model and experimental groups

Female Wistar rats (200 ± 15 g; n=150) were provided by the Animal Center of Shandong University (Jinan, China). Animal experiments were performed in accordance with the International Guiding Principles for Animal Research provided by the Council for International Organizations of Medical Sciences (CIOMS), and procedures were approved by the Animal Ethical and Welfare Committee of Jinan Central Hospital.

Rats were anesthetized by intraperitoneal injection of 10% chloral hydrate (3mL/kg) and fixed in a prone position. The dorsal skin and muscles were retracted, then the spinous process was identified and separated, and the T10 lamina was carefully severed to expose the spinal cord. A bulldog clamp was used to hit the spinal cord for 30 s. Observation of a spastic movement of the rat's tail and paralysis of the lower limb after trembling indicated successful spinal cord injury. For sham operation, rats were anesthetized and a laminectomy was performed at T10 [38].

The rats were randomly divided into 5 groups (6 rats per group): 1) sham; 2) PBS; 3) ADSC; 4) ADSC + SB431542; and 5) SB431542. Three days after injury, SCI rats received PBS, ADSC, and SB431542 treatments. In the ADSC-treated groups, a total of 5×10^5^ cells in 10 μL PBS were administered via two injections applied 2 mm rostral and 2 mm caudal to the lesion using a microliter syringe. SB431542 (1 mg/kg) was administered by intraperitoneal injection once a day for 7 days. At various time points (3, 7, 14, and 28 days after treatments, i.e. PBS, ADSC and/or SB431542), 6 rats were sacrificed by euthanasia with an overdose of 10% chloral hydrate. Blood was extracted directly from the heart and centrifuged to collect sera for further analysis. At injury sites, ~10-mm thick spinal cord tissue sections were harvested for further analysis.

### Behavioral tests

On days 3, 7, 14, 21, and 28 after drug treatment and/or ADSC transplantation, locomotor recovery was evaluated using the test developed by Basso et al. [39]. Hindlimb joint movement, paw placement, weight support, and forelimb-hindlimb coordination were ranked on a scale from 0–21 [40].

### Histological analysis

On the 3^rd^ day after drug treatment and/or ADSC transplantation, 3 rats in each group were deeply anesthetized with an intraperitoneal injection of 10% chloral hydrate. Spinal cord tissues were fixed in 4% paraformaldehyde at 4° C for 48 h, dehydrated, embedded, cut into 3-μm transverse sections, and processed for H&E and Nissl staining [41].

### Statistical analyses

Data are presented as the mean ± standard deviation. Experimental results were analyzed by two-tailed t-test or one-way analysis of variance (ANOVA). SPSS 22.0 software (SPSS Inc., Chicago, IL, USA) was used for all statistical analysis, and *P* < 0.05 was considered significant.

## Supplementary Material

Supplementary Figures

Supplementary Table 1
