# Supplementary material for: Adipose-derived stromal cells improve functional recovery after spinal cord injury through TGF-β1/Smad3/PLOD2 pathway activation
Source: Aging (Albany NY). 2021 Jan 20;13(3):4370–87. doi: 10.18632/aging.202399 (PMC7906172; doi:10.18632/aging.202399)
Supplement: Supplementary Figures [file aging-13-202399-s001.pdf]

SUPPLEMENTARY FIGURES

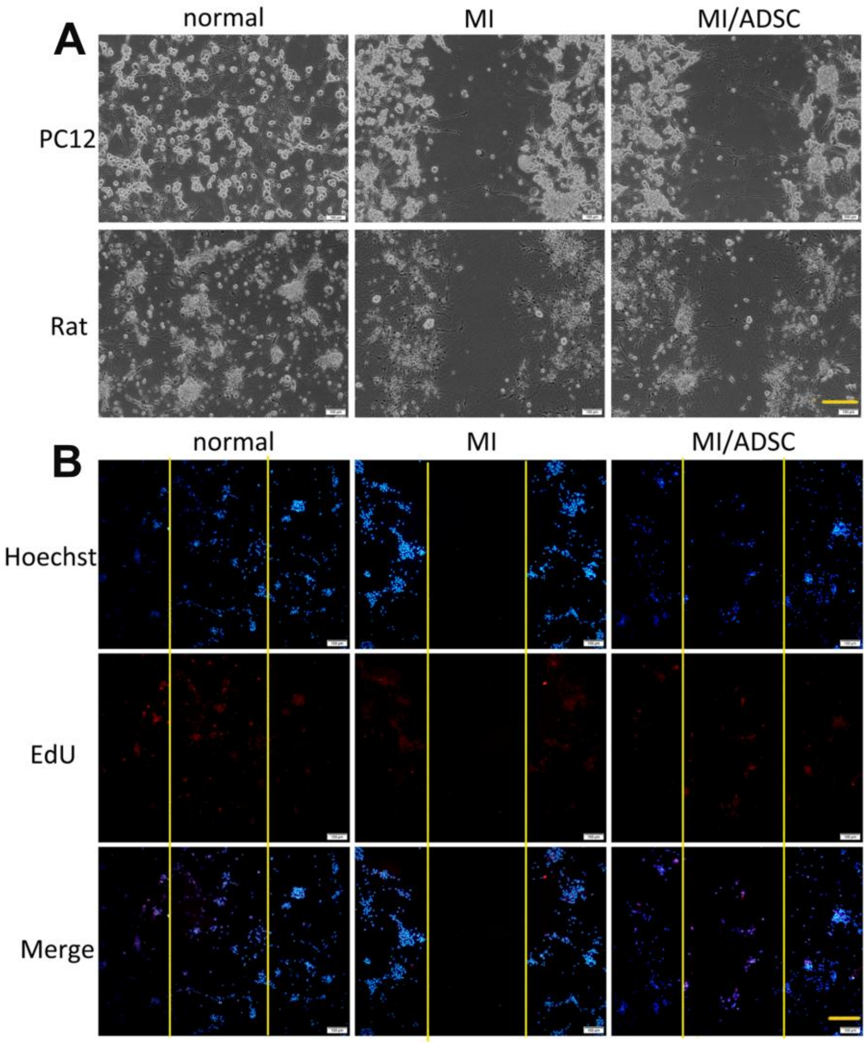

Supplementary Figure 1. ADSCs promote neuronal recovery *in vitro*. (**A**) Light microscopy results showed that wound closure rates were significantly higher in cells co-cultured with ADSCs. Scale bar: 100µm. Rat: rat cortical neurons. (**B**) EdU results showed that co-cultured ADSCs enhanced the proliferation of rat cortical neurons. Scale bar: 100µm. MI: mechanical injury.

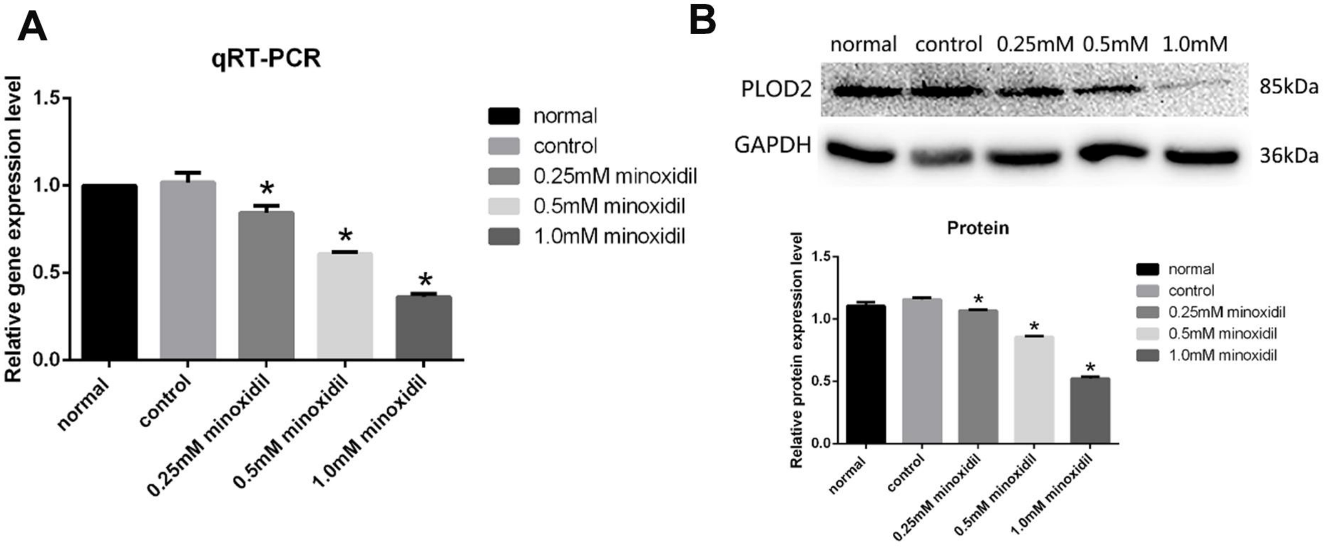

Supplementary Figure 2. Screen the optimal concentration of minoxidil to suppress the expression of PLOD2 in PC12 cells. (**A**) qRT-PCR analysis showed that the inhibitory effect of PLOD2 was enhanced with the increase of minoxidil concentration. (**B**) Western blot analysis. Results presented as mean ± SD and evaluated with one-way ANOVA. * *P< 0.05.*
