# Supplementary material for: Adipose-derived stromal cells improve functional recovery after spinal cord injury through TGF-β1/Smad3/PLOD2 pathway activation
Source: Aging (Albany NY). 2021 Jan 20;13(3):4370–87. doi: 10.18632/aging.202399 (PMC7906172; doi:10.18632/aging.202399)
Supplement: Supplementary Table 1 [file aging-13-202399-s002.pdf]

SUPPLEMENTARY TABLE

Supplementary Table 1. Primer sequence for qRT-PCR.

Primer name	Forward primer sequence	Reverse primer sequence
β-actin	GCCGGGACCTGACAGACT	TGGCCATCTCTTGCTCGA
TGF-β1	GAAACGGAAGCGCATCGA	TGGCGAGCCTTAGTTTGGA
PLOD2	TCCCGTGGATTGGAAGGAAA	ACGGCTGTCATGATGCTTTC
GFAP	TCTGCCCAGTGAGTAAAGGTGA	GGTGTGGAGTGCCTTCGTATTA
NSE	AACTTCCGGAATCCCAGTGT	AGGTGAGTCGAGGTGTTCTG
MAP2	CATTTTGGTGCTGATCTCTCT	AGCTGGCCTTCTCCACAC
GAP43	TCCTCTCCTGTCCTGCTCAC	TCGCCATAACAACACCAAGA
